# Sensory Feedback Interferes with Mu Rhythm Based Detection of Motor Commands from Electroencephalographic Signals

**DOI:** 10.3389/fnhum.2017.00523

**Published:** 2017-11-01

**Authors:** Maximilian Hommelsen, Matthias Schneiders, Christian Schuld, Philipp Keyl, Rüdiger Rupp

**Affiliations:** Experimental Neurorehabilitation, Spinal Cord Injury Center, Heidelberg University Hospital, Heidelberg, Germany

**Keywords:** electroencephalography, brain-computer interface, mu rhythm, alpha rhythm, sensory feedback, motor execution, sensorimotor integration, motor rehabilitation

## Abstract

**Background:** Electroencephalogram (EEG)-based brain-computer interfaces (BCI) represent a promising component of restorative motor therapies in individuals with partial paralysis. However, in those patients, sensory functions such as proprioception are at least partly preserved. The aim of this study was to investigate whether afferent feedback interferes with the BCI-based detection of efferent motor commands during execution of movements.

**Methods:** Brain activity of 13 able-bodied subjects (age: 29.1 ± 4.8 years; 11 males) was compared between a motor task (MT) consisting of an isometric, isotonic grip and a somatosensory electrical stimulation (SS) of the fingertips. Modulation of the mu rhythm (8–13 Hz) was investigated to identify changes specifically related to the generation of efferent commands. A linear discriminant analysis (LDA) was used to investigate the activation pattern on a single-trial basis. Classifiers were trained with MT vs. REST (periods without MT/SS) and tested with SS and vice versa to quantify the impact of afferent feedback on the classification results.

**Results:** Few differences in the spatial pattern between MT and SS were found in the modulation of the mu rhythm. All were characterized by event-related desynchronization (ERD) peaks at electrodes C3, C4, and CP3. Execution of the MT was associated with a significantly stronger ERD in the majority of sensorimotor electrodes [C3 (*p* < 0.01); CP3 (*p* < 0.05); C4 (*p* < 0.01)]. Classification accuracy of MT vs. REST was significantly higher than SS vs. REST (77% and 63%; *p* < 10^-8^). Classifiers trained on MT vs. REST were able to classify SS trials significantly above chance even though no motor commands were present during SS. Classifiers trained on SS performed better in classifying MT instead of SS.

**Conclusion:** Our results challenge the notion that the modulation of the mu rhythm is a robust phenomenon for detecting efferent commands when afferent feedback is present. Instead, they indicate that the mu ERD caused by the processing of afferent feedback generates ERD patterns in the sensorimotor cortex that are masking the ERD patterns caused by the generation of efferent commands. Thus, processing of afferent feedback represents a considerable source of false positives when the mu rhythm is used for the detection of efferent commands.

## Introduction

Brain-computer interfaces (BCI) are systems that allow controlling computers or external devices directly through changes in brain activity, serving as a technical bypass for a dysfunctional neuromuscular system (Nicolas-Alonso and Gomez-Gil, 2012). Much of the pioneering work on BCIs was centered on the attempt to enable patients with a locked-in syndrome following amyotrophic lateral sclerosis to communicate and interact with their environment through focused attention (Wolpaw et al., 1991; Kubler et al., 2001). The technology of non-invasive BCIs based on electroencephalographic (EEG) recordings has reached a level of technological and usability readiness for independent application by end users and their caregivers. Nowadays, studies have demonstrated the feasibility of BCIs to control robotic arms (Onose et al., 2012), arm orthotics (Pfurtscheller et al., 2003) combined with functional electrical stimulation (FES) (Rohm et al., 2013) or electrical wheelchairs (Galan et al., 2008) in order to compensate the loss of basic motor functions in individuals suffering from severe motor disabilities.

In applications where motor functions are directly substituted by assistive devices such as robot arms or neuroprostheses, it is desirable to establish an intuitive BCI control with brain signals originating in the motor cortex. The most prominent changes in brain activity associated with the generation of motor behavior can be detected in the area of the sensorimotor cortex when non-invasive recordings are performed (Arroyo et al., 1993). The mu rhythm (8-13 Hz) is such a characteristic oscillation measured in the EEG and can be described as a sensorimotor cortical equivalent to the generic occipital alpha rhythm. The mu rhythm reflects the modulation or transfer of sensorimotor information in the process of motor preparation and execution (Pfurtscheller et al., 1996; McFarland et al., 2000). Likewise, it is increased during physical rest, i.e., in a state of sensorimotor cortical idling and attenuated with onset of physical activity (Kuhlman, 1978; Babiloni et al., 1999). Furthermore, alterations of the mu rhythm occur in relation to the somatotopic organization of the sensorimotor cortex permitting the distinction of brain activity associated with movement of different limbs (Yuan et al., 2010). Most important, however, it has been demonstrated that people are able to attenuate their mu rhythm merely through mental simulation of motor actions without actually performing the movements after some practice (Yuan and He, 2014). Thus, the mu rhythm is a suitable candidate to realize natural and intuitive BCI control as it originates in the sensorimotor cortex and its modulation is directly associated with the user’s intention to perform motor actions. Indeed, the analysis of the mu rhythm has been implemented in various forms during the attempt to establish BCI control (Wolpaw and McFarland, 2004; Pfurtscheller et al., 2006; Yuan and He, 2014). However, attenuation of the mu rhythm is not exclusively related to the generation of motor commands. Instead, pure somatosensory stimulation in absence of movements or motor imageries also leads to attenuation of the somatotopic mu rhythm (Pfurtscheller, 1989; Schnitzler et al., 1995; Cheyne et al., 2003). Thus two different components involved in the generation of physiological motor control are indicated by an attenuation of the mu rhythm: (1) the imagination and execution of motor actions in the motor cortex and (2) the processing of afferent feedback in the sensory cortex.

In recent times, there are considerations that BCIs could be used as rehabilitative rather than assistive technology (Daly and Wolpaw, 2008), e.g., to support upper limb motor recovery in stroke patients (Ang et al., 2010; Prasad et al., 2010). After stroke, the brain undergoes complex pattern of injury-related reorganization, compensating some of the structural damage, eventually leading to spontaneous recovery of motor function in some patients (Hallett, 2001; Cauraugh and Summers, 2005; Grefkes and Ward, 2014). However, this intrinsic capacity of the brain to compensate damage is limited (Grefkes and Ward, 2014) and a full motor recovery is more an exception than a rule. Stroke rehabilitation therapies are mainly based on the concept of motor learning that requires patients to perform specific motor tasks (MTs; Krakauer, 2006). Most patients are limited in their abilities to execute a movement properly or in the worst case, are unable to perform any movement. In this regard, BCIs offer the possibility to identify the patient’s movement intention without movements being present. The BCI-detection of the movement intention enables the possibility for motor training through an orthotic or FES device that moves the limb according to the movement intention, simultaneously generating sensory feedback, closing the sensorimotor loop (Gomez-Rodriguez et al., 2011; Soekadar et al., 2015) and therewith promoting the intrinsic capacities of neuroplastic reorganization through motor learning (Nudo et al., 1996; Krakauer, 2006). Several studies investigated the user acceptance concerning invasive and non-invasive BCIs, concluding that non-invasiveness is a high-priority design requirement even in the development of assistive neuroprosthetic devices (Collinger et al., 2013; Waldert, 2016). It can be expected that end user and health professionals’ acceptance is even lower for invasive BCIs when the system is used as a rehabilitative device within the limited and usually foreseeable time schedule of motor training after central nervous system injury. Thus, rehabilitative neuroprosthetic devices will most likely face the challenge to decode the user’s motor intention on the basis of non-invasively recorded brain signals with poor spatial resolution.

The success of such closed-loop rehabilitative neuroprosthetic devices is dependent on whether the user’s movement intention can be reliably decoded from the sensorimotor cortex when somatosensory feedback is processed simultaneously. This aspect deserves particular attention for several reasons: Even though sensory cortex and motor cortices are distinct anatomical regions, they share the same somatotopic organization and have an important functional relationship. The realization of complex and smooth limb movements relies heavily on the integration of sensory information to permit the dynamical adjustment of the concurrent movement state (Peterka, 2002; Scott, 2004). If this sensory feedback is absent, as in patients suffering from large fiber sensory neuropathies, a disastrous effect on motor control can be observed (Cole, 1995; Ghez et al., 1995). Thus, movements are inevitably associated with considerable amounts of sensorimotor integration and require a concurrent activation and interaction of the motor and sensory cortices. If motor imagery is used to initiate and control movements that generate sensory feedback in a closed-loop manner, it raises the question whether the activation of the sensory cortex interferes with the attempt to detect the user’s motor commands encoded in the activation of the motor cortex. Given that both, the imagery and execution of motor actions and the processing of afferent feedback lead to an attenuation of the mu rhythm in the sensorimotor cortex, it is unclear whether it can be clearly distinguished if the attenuation represents activation of the efferent pathways, i.e., execution and imagery of motor actions in the motor cortex or the processing of afferent information in the sensory cortex.

The aim of the following study is to find out if there are distinct differences in the modulation of the mu rhythm that are attributed to the generation of efferent commands. Furthermore, the brain activation patterns were investigated with a classification algorithm to evaluate whether it is desirable to use the mu rhythm as signal to detect the generation of efferent motor commands when sensory feedback is processed simultaneously. For this purpose, brain activity was recorded during performance of a MT that consisted of an isometric grip and a somatosensory electrical stimulation (SS) of the fingertips. In this way, it was possible to investigate (1) how a combined activation of sensory and motor cortex influences the mu rhythm and (2) how solely an activation of the sensory cortex without motor cortex influences the mu rhythm.

## Materials and Methods

### Participants

Thirteen able-bodied, right-handed subjects (age: 29.1 ± 4.8 years; 2 female, 11 male) without known history of neurological or musculoskeletal disorders participated in the study approved by the ethical committee of the Heidelberg University (S-016/2014). All subjects gave their written informed consent prior to the experiment in accordance with the Declaration of Helsinki. Right-handedness was determined before the experiment using the Hand-Dominanz-Test (Steingrüber and Lienert, 1971). Participants were seated in a comfortable chair with both arms fully supported by the armrests and placed in front of a computer screen in ∼1 m distance and EEG was recorded. Subjects were advised to focus on the screen, leave their arms in the resting position on the armchairs and avoid voluntary movements with their hands and body if not stated otherwise throughout the experiment.

### Data Acquisition

Brain signals were acquired using a g.GAMMAcap with 64 active electrodes (g.tec medical engineering GmbH, Schiedlberg, Austria) arranged in the international 10–10 system covering the entire scalp. The ground electrode was placed slightly anterior between the Fz and F2 electrodes. The reference electrode was placed on the mastoid part of the left temporal bone. Impedances of the electrodes were kept below 30 kΩ and checked after each block. Signals were recorded at a sampling rate of 512 Hz and amplified with a multichannel EEG-amplifier (g.HIamp, g.tec medical engineering GmbH, Schiedlberg, Austria), band-pass filtered from 0.1 to 100 Hz and notch filtered at 50 Hz. Grip force was measured with five piezoresistive force sensors (FlexiForce A201 Sensor; Tekscan Inc., South Boston, MA, United States). Sensors were attached to a cylinder (height = 120 mm, Ø = 80 mm) and arranged in a way that each of the subject’s fingertips covered exactly one sensor during a natural cylinder grip. The cylinder was fixed to the table in front of the computer screen. Sensors were connected to the g.HIamp amplifier and simultaneously recorded with the EEG signals at a sampling rate of 512 Hz. A dome of epoxide resin (height: 2 mm, surface: ∼1 cm^2^) was glued to the force sensitive area of each sensor to achieve a uniform distribution of forces on the force sensitive area (Jensen et al., 1991). Furthermore, it facilitated the subject’s accurate finger placement for less variability of finger positioning and higher accuracy of the measurements.

### Functional Electrical Stimulation

Functional electrical stimulation was used to generate a uniform superficial somatosensory perception on the subjects’ fingertips. FES was performed with a MotionStim8 (MEDEL GmbH, Hamburg, Germany). Neuroline 700 surface electrodes (Ambu GmbH, Bad Nauheim, Germany) with a contact area of ∼2.6 cm^2^ were attached to the subject’s right hand fingertips. The corresponding return hydrogel electrode (KRAUTH+TIMMERMANN GmbH, Hamburg, Germany) with a surface of ∼44 cm^2^ was placed on the subject’s right proximal forearm close to the fossa cubitalis. The stimulation was executed with a current of 1 mA and a pulse width of 50 μm (rectangular charged-balanced-pulses with 100 μs pulse pause between stimulation and charge balancing pulses). Stimulation frequency was set to 60 Hz or 90 Hz depending on the specific experimental condition.

### Experimental Design

**FIGURE 1:**
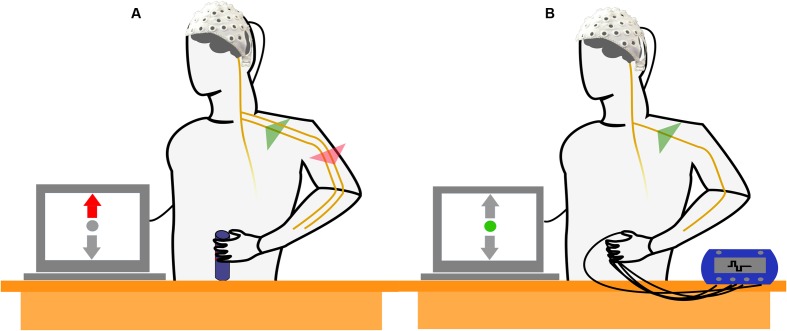
Schematic drawing of the experimental setup and the experimental conditions. **(A)** The subject performs an isometric, isotonic grip around a cylinder. The motor task (MT) reflects a combined activation of motor and sensory cortices as efferent motor commands are generated and sensory feedback is processed simultaneously. **(B)** Subject is not involved in a MT but electrical stimulation of the fingertips is applied. The electrical stimulation simulates a sensory perception that resembles the superficial sensory input during the isometric grip in absence of the generation of efferent motor commands.

Two different experimental tasks were compared within the paradigm. In MT blocks, subjects were instructed to perform a right-handed MT with the intention to observe a combination of motor cortical activation (generation of efferent commands) and activation of the sensory cortex (processing of afferent feedback) in the EEG. The MT required the subjects to produce and maintain a specific amount of force with an isometric grip around a cylinder based on a visual feedback indicating the applied force (**Figure 1A**). In SS blocks, subjects kept their arm and hand in a resting position and no MT was executed. Instead, electrical stimulation of the fingertips was performed with the intention to generate a sensory perception that resembled the superficial sensory input perceived during execution of the grip in absence of any motor related activity (**Figure 1B**). Subjects were instructed to avoid any kind of voluntary movement during application of FES.

The tasks were carried out with two different intensities: *low* and *high*. In MT blocks, low and high indicated the amount of isometric grip force that was required to successfully perform the MT. In SS blocks, this referred to the low (60 Hz) and high (90 Hz) electrical stimulation frequency. The MT will be referred to as MT_LOW_ and MT_HIGH_ for execution with low and high grip force. The same notation will be used for SS trials, i.e., SS_LOW_ and SS_HIGH_ for low and high stimulation frequency. In addition, each block comprised REST trials that were identical to the other trials except no MT was performed and no electrical stimulation pulses applied. The REST trials from MT blocks and SS blocks were used as individual baselines for MT trials and SS trials.

Subjects performed at least 50 successful trials of each experimental condition, summing up to more than 300 trials (including REST trials) in total. In average, trials lasted approximately 12 s and the complete experiment was finished after approximately 90 min. Each subject performed six blocks with at least 50 trials. The sequence of blocks and trials was randomized with the constraint that SS and MT trials were not mixed within the same block. REST trials were equally present within the sum of all MT and SS blocks. Incorrect trials (e.g., subject failed to maintain the required force or executed MT during REST trial were repeated by shuffling them back into the remaining sequence of trials within the block.

### Trial Structure

**FIGURE 2:**
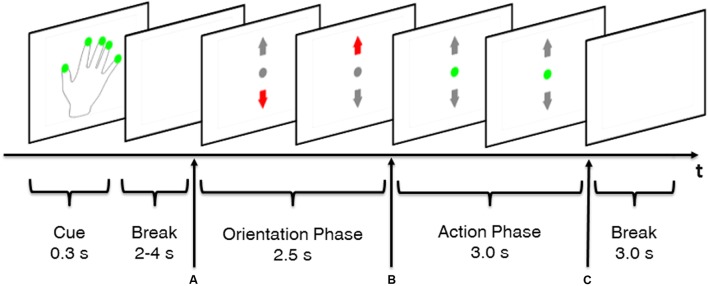
Time course of a MT trial. Green dots on the fingerprints of the cue hand indicated that a non-REST trial was performed, i.e., execution of the MT. A random break taking between 2 and 4 s gave the subject enough time to place his fingers on the force sensors that were attached to the cylinder. Appearance of visual feedback **(A)** served as GO cue to apply grip force. An orientation phase gave the subject time to adapt his grip force to the predefined target force. Grip force was evaluated during the action phase **(B,C)** to determine whether the subject’s force was in the target range. Disappearance of the visual feedback **(C)** served as STOP cue. Each trial was followed by a 3.0 s inter-trial break.

The structure of the trials will be illustrated using a MT trial as example (**Figure 2**). Trials started with a cue (0.3 s) indicating whether or not a REST trial was going to occur. A hand with green dots on the fingertips indicated that execution of MT was required whereas a plain hand without green dots indicated a REST trial. However, subjects received no information whether a MT_LOW_ or MT_HIGH_ trial was going to occur. The cue was followed by a random break sequence of 3–4 s which provided enough time for the subjects to switch their hand from the resting position into the grip position with their fingers loosely placed on the force sensors. Appearance of the visual feedback screen (**Figure 2A**) served simultaneously as GO cue for execution of the MT. Excessive grip force was indicated by a red upward arrow whereas a too low force was indicated by a red downward arrow in the visual feedback. Display of a green dot indicated that the applied grip force matched the required target force. Target intervals were specified around the target forces. These target intervals were defined as an average force of 2.5 N ± 20% per finger for MT_LOW_ trials and an average of 6.5 N ± 10% per finger for MT_HIGH_ trials. In the first 2.5 s after appearance of the visual feedback (**Figure 2A**) subjects were given time to adapt their grip force with the target force interval. After this orientation phase, grip force was evaluated for 3 s to determine the success of the trial (**Figures 2B,C**). The MT was completed successfully if the subject’s grip force remained within the target force interval for 80% of the time of the 3 s action phase. After the 3 s action phase, the visual feedback disappeared simultaneously serving as a STOP cue (**Figure 2C**). In order to ensure that the visual stimulus was consistent across experimental conditions, a visual feedback sequence randomly chosen from a group of 50 pre-recorded MT trials was displayed during SS and REST trials. SS trials showed a small deviation from MT trials as electrical stimulation started at the start of the action phase (**Figure 2B**). Thus, orientation phase only consisted of a visual feedback and did not include application of FES. Furthermore, the break after cue phase was reduced to 2 s as no change in hand position was required.

### Data Preprocessing

Electroencephalogram recordings were band-pass filtered from 0.5 to 40 Hz and notch-filtered at 36–39 Hz due to an unknown source of contamination at 37.5 Hz present only in the room where the experiments took place. Signals were down-sampled to 128 Hz for the sake of faster post-processing. All EEG recordings were inspected visually and electrodes with too high impedance (>30 kΩ) or poor signal quality were removed completely from further analysis. Time segments containing electromyographic or movement-related artifacts (mastication, sudden head movements, changing posture) were removed from the affected electrodes individually. Electrodes in which more than 50% of the data points were removed were excluded completely from the subsequent analysis. Three sequences of eye movements (blinks, vertical, and horizontal), each generating a maximum of electrooculographic activity, were recorded prior to the experiment. They were used as template for the automatic recognition and removal of electrooculographic activity in the experimental EEG signals based on the interference subtraction method described by Parra et al. (2005). Last, signals were referenced to the Common Average Reference (McFarland et al., 1997).

### Data Analysis

All successful trials were extracted from the datasets and a time interval of 2 s length, starting 0.5 s after beginning of the action phase and ending 0.5 s before the end of the action phase (**Figures 2B,C**), was defined as signal period of interest for subsequent analysis and classification. This time interval was chosen as the subject performs an isometric grip with a constant amount of force during that period. It was expected that the somatosensory feedback perceived during that interval matches best with the perception evoked during SS using FES. Time-frequency representations were computed for each electrode on a single-trial basis by applying the Fast Fourier Transform (FFT) on the 2 s time interval. A maximum of 50 intervals per subject per experimental condition were obtained according to this scheme. The activation of electrode *E* was calculated by averaging the amplitude of all frequency bins *n* = 1…*M* in the frequency band and all trials *k* = 1…*L* of the specific condition:

$$E_{q}=\frac{1}{M}\frac{1}{L}\overset{M}{\underset{n=1}{\Sigma }}\overset{L}{\underset{k=1}{\Sigma }}x_{nk}$$

with *q* = 1…*P* denoting the number of electrodes and *x*_nk_ the amplitude in the frequency spectrum at frequency bin *n* in trial *k*. The relative power change at electrode *E* was calculated according to:

$$\%{Power}_{E}=\frac{\exp_{E}-{rest}_{E}}{{rest}_{E}}$$

with *exp_E_* and *rest_E_* denoting the activity of electrode *E* during experimental condition and REST, respectively. Using Eqs (1) and (2), the relative power change in brain activity %*Power_E_* was determined for each electrode and experimental condition and averaged across all subjects. Positive power changes will be referred to as event-related synchronization (ERS) whereas negative changes will be referred to as event-related desynchronization (ERD) in accordance with the common notation described by Pfurtscheller and Lopes da Silva (1999). Results were visualized for the alpha (8–13 Hz) and the beta band (14–30 Hz) as scalp maps.

### Classification on a Single-Trial Basis

The classification of single-trials was performed using a linear discriminant analysis (LDA) classifier (Fisher, 1936). A rather simple feature vector, consisting of 20 spatial features, was used for classification. Each feature represents neural activity captured at one of the twenty electrodes covering bilateral sensorimotor and central areas (CF3, FC1, FCz, FC2, FC4, C4, C2, Cz, C1, C3, CP3, CP1, CPz, CP2, CP4, P4, P2, Pz, P1, P3; based on Galán et al., 2015). Features were calculated by averaging the amplitude of all frequency bins in the alpha band derived from the time interval of interest for each trial and specific electrode. Based on numerous studies that indicate the importance of beta oscillations during movement execution and object grasping (McFarland et al., 2000; Kilavik et al., 2013; Zaepffel et al., 2013), all classifications were performed a second time with another feature vector. This vector included the 20 features derived from the alpha band plus 20 additional features derived from the beta band, calculated in an identical way to the alpha band features. In this way, it was possible to examine whether the beta band contributes additional information that might improve the detection of efferent motor commands.

Regular classification performance was assessed for all experimental conditions subject-wise using four distinct LDA classifiers trained in a binary classification task to distinguish (1) MT_LOW_ vs. REST (2) MT_HIGH_ vs. REST (3) SS_LOW_ vs. REST and (4) SS_HIGH_ vs. REST. These four classifiers will be referred to as regular classifiers (RC) because they were trained and tested on the same dataset using a five-fold cross-validation and thus reflect the general classification performance. Since all RCs performed a classification of an experimental condition against REST, they will be referred to as MT_LOW_, MT_HIGH_, SS_LOW_, and SS_HIGH_ without specifically mentioning the REST condition for the convenience of shorter notation.

In a second classification experiment, we attempted to target the problem whether the classification of MT trials is solely driven by the discriminatory properties of EEG signals that originate in the motor cortex during ME or if it might be driven by a combination of efferent-related and sensory feedback-related components in the EEG signal. Classifications were repeated but the classifiers’ performances were assessed with trials from the opposite class, meaning that MT classifiers were tested with SS trials and SS classifiers were tested with MT trials. This process will be denoted as cross-classification and the respective classifiers as cross-classifiers (CC). An example: the classifier was trained to distinguish MT_LOW_ vs. REST trials. Instead of evaluating the classifier’s performance with a dataset comprising MT_LOW_ and REST trials, the performance was now evaluated with a dataset comprising SS_LOW_ vs. REST trials or SS_HIGH_ vs. REST trials. Thus, the performance of both MT classifiers (MT_LOW_ and MT_HIGH_) was tested with both types of SS trials (SS_LOW_ and SS_HIGH_) and vice versa. CCs will be described by mentioning the class on which they were trained first and secondly the class with which they were tested. Again, REST trials will be omitted in the notation. As example: the cross-classifier MT_LOW_/SS_HIGH_ was trained with dataset comprising MT_LOW_ vs. REST trials and tested with a dataset comprising SS_HIGH_ vs. REST trials.

### Software and Statistical Analysis

Data processing, analysis, and classification were performed using MATLAB R2014a (MathWorks, Inc., Natick, MA, United States). Signal recording and presentation of the paradigm was integrated into a custom-made MATLAB Simulink model. The results from the spatial activation pattern are given as the relative power change from baseline ± standard deviation (SD). The classifiers were trained and tested using the *fitcdiscr()/predict()* from the Statistics and Machine Learning Toolbox. Topographical plots were created using a custom-made MATLAB function. Significant changes in electrode activity and classification accuracy were calculated using a two-way analysis of variance (ANOVA). The threshold for statistical significance was set to α ≤ 0.05. α values ≤ 0.10 will be denoted as trend. The *post hoc* analysis was performed using the Tukey–Kramer method.

Performance of the classifiers was evaluated in terms of classification accuracy, i.e., the percentage of correctly predicted class memberships amongst all classified trials. Classification accuracy was considered significant if the percentage of correctly classified trials exceeded chance level. As classification error can be assumed to follow a binomial cumulative distribution (Combrisson and Jerbi, 2015), classification accuracy was considered significantly above chancel level (*p* ≤ 0.05) when the percentage of correctly predicted trials exceeded approximately 59%. Classification results are reported as mean ± standard error of the mean (SEM) in the text and as box plots in the figures. Central lines in the box plots indicate the median, while diamonds represent the mean.

## Results

### Motor Task Performance

On average, subjects completed the MT successfully in 86.4 ± 8.8% of MT_LOW_ trials and in 81.6 ± 8.5% of MT_HIGH_ trials. The majority of subjects (9 out of 13) performed the MT with low grip force better than the MT requiring high grip force. However, this difference in performance was not statistically significant but indicated a trend (*p* = 0.09).

### Signal Quality

On average, 79.5 ± 10.1% of the recorded data point survived preprocessing steps and were included in the analysis. Frontal and temporal channels were affected stronger by physiological artifacts like eye blinks, mastication or head movements than other electrodes. In contrast, electrodes in the center of the scalp provided a better signal quality and were only rarely affected by artifacts or technical interferences. Electrodes FP1 and F6 were excluded from the analysis completely since they exhibited extremely poor signal quality in almost all subjects and less than 50% of the data points survived preprocessing steps.

### Analysis of Power Changes in the Alpha and Beta Band

**FIGURE 3:**
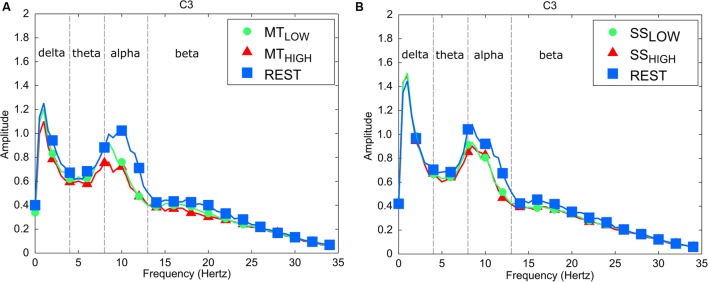
Frequency spectra depicting characteristic power changes around the contralateral sensorimotor cortex (C3) averaged over all subjects. Spectrum with blue squares depicts the rest condition. Spectrums with green circles and red triangles depict the experimental conditions performed with low and high intensity, respectively. **(A)** Execution of the MT leads to a significant mu event-related desynchronization (ERD) in comparison to the rest condition for low and high task intensities. There was no significant difference in mu ERD between execution of the MT with low and high intensity. **(B)** Sensory stimulation (SS) leads to a significant mu ERD compared to the rest condition. The mu ERD caused by SS was smaller than the mu ERD caused by the MT. There was no significant difference in mu ERD between SS with low and high intensity.

The frequency spectra in **Figure 3** illustrate the power changes during execution of the MT (left), SS of the fingertips (right) vs. the respective REST condition recorded around the contralateral sensorimotor cortex (C3) averaged over all trials and subjects. As expected, execution of the MT and SS were associated with a prominent decrease in power spectral density in the alpha band in comparison to the REST condition. In comparison, alterations in the beta band were extremely small during execution of the MT and SS. Similar alpha band power changes were observed in all bilateral sensorimotor electrodes with varying degrees of manifestation (data not shown).

#### Spatial Activation Pattern in the Alpha Band

**FIGURE 4:**
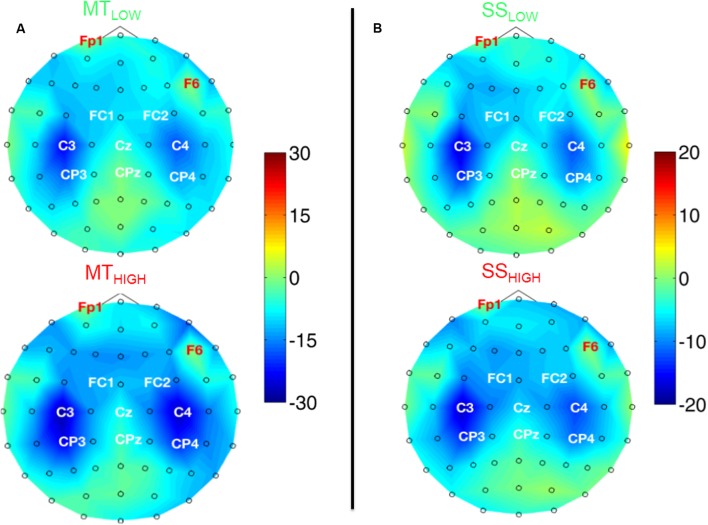
Scalp maps depicting power changes in the alpha band during execution of the MT (subfigure **A**) and sensory stimulation of the fingertips (SS; subfigure **B**) relative to rest and averaged over all subjects. Event-related synchronization (ERS) is encoded in red and desynchronization (ERD) in blue. Channels denoted with red letters were removed from the analysis and set to neutral in the plots. All experimental conditions were characterized by highly similar spatial configurations covering bilateral sensorimotor areas with peak ERDs around the contralateral motor cortex (C3), ipsilateral motor cortex (C4), and the contralateral centro-parietal cortex (CP3). Execution of the MT lead to a significantly stronger ERD in these channels compared to sensory stimulation (note the scaling of the color bars). There were no significant differences within the ERDs caused by the MT with low and high intensity or SS with low and high intensity.

**Figure 4** shows the topographical distribution of alpha power changes relative to the baseline (REST) averaged over all subjects for all experimental conditions. The scalp maps revealed ellipsoid ERD spots covering bilateral sensorimotor cortices (i.e., mu rhythm) primarily including fronto-central (FC), central (C), and centro-parietal (CP) electrodes. These spatial pattern were preserved in all experimental conditions and characterized by a peak ERD at C3, followed by a smaller ERD in CP3 and FC3 (pre-motor cortex) in the contralateral hemisphere. A symmetrical spatial configuration was detected at the ipsilateral hemisphere with a peak ERD at C4, followed by a smaller ERD at CP4 and FC4. The largest spectral power decreases relative to the baseline were always detected in the same three electrodes in all experimental conditions (**Table 1**), localized around the contralateral (C3) and ipsilateral sensorimotor cortex (C4) as well as the contralateral centro-parietal cortex (CP3).

**Table 1:** Decrease in alpha power in sensorimotor electrodes given as relative change (%) from baseline (REST).

	Contralateral			Ipsilateral		
		
Electrode	C3	CP3	FC3	C4	CP4	FC4
MT_LOW_	23.8	17.8	11.9	18.1	13.7	10.6
MT_HIGH_	28.9	24.6	14.3	26.9	21.0	15.5
SS_LOW_	17.2	14.0	9.4	12.3	10.3	5.6
SS_HIGH_	18.6	14.5	10.9	12.6	9.9	6.8


Execution of the MT_LOW_ lead to a significant reduction of alpha band power in FC3 (*p* < 0.05), C3 (*p* < 0.001), CP3 (*p* < 0.05), C4 (*p* < 0.01), and CP4 (*p* < 0.01) compared to REST. Electrode FC1 indicated a trend (*p* = 0.1). Execution of the MT_HIGH_ lead to a significant reduction of alpha band power in FC3 (*p* < 0.01), FC1 (*p* < 0.05), FC4 (*p* < 0.05), C3 (*p* < 0.001), C1 (*p* < 0.05), C2 (*p* < 0.05), C4 (*p* < 0.0001), and CP3 (*p* < 0.001). Electrodes FCz (*p* = 0.07), FC2 (*p* = 0.07), CP2 (*p* = 0.08), P3 (*p* = 0.09), P3 (*p* = 0.1) indicated a trend. In contrast, a significant reduction of alpha band power was only observed in electrode C3 (*p* < 0.05) during SS_LOW_ and SS_HIGH_. Electrode CP3 indicated a trend (*p* = 0.08 and *p* = 0.07) in both conditions whereas electrode C1 indicated a trend only during SS_HIGH_ (*p* = 0.08).

There were no significant differences in alpha band power between both MT conditions (**Figure 4A**) and no significant differences between both SS conditions (**Figure 4B**) in all of the 20 bilateral sensorimotor electrodes that were included in the analysis. However, a comparison of power changes of MT_LOW+HIGH_ vs. SS_LOW+HIGH_ revealed a significantly reduced alpha band power in FC3 (*p* < 0.05), FC1 (*p* < 0.01), FCz (*p* < 0.01), FC2 (*p* < 0.01), FC4 (*p* < 0.01), C3 (*p* < 0.01), C1 (*p* < 0.05), Cz (*p* < 0.05), C2 (*p* < 0.01), C4 (*p* < 0.01), CP3 (*p* < 0.05), and P3 (*p* < 0.001) during execution of the MT compared to SS. Electrode CP4 indicated a trend (*p* = 0.06).

#### Spatial Activation Pattern in the Beta Band

**FIGURE 5:**
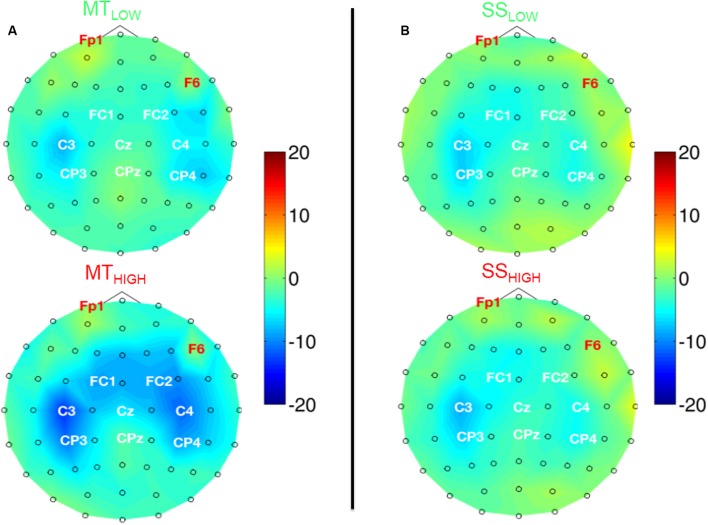
Scalp maps depicting power changes in the beta band during execution of the MT (subfigure **A**) and sensory stimulation of the fingertips (SS; subfigure **B**) relative to rest and averaged over all subjects. Event-related synchronization (ERS) is encoded in red and desynchronization (ERD) in blue. Channels denoted with red letters were removed from the analysis and set to neutral in the plots. All experimental conditions were characterized by a peak ERD around the contralateral motor cortex (C3). Execution of the MT with high intensity showed some involvement of the contralateral centro-parietal cortex (CP3) and a spread toward fronto-central areas. SS with low and high intensity showed a lateralization toward the contralateral motor cortex (C3) and only little involvement of the ipsilateral sensorimotor areas. There were no significant differences within the ERDs caused by the MT with low and high intensity or SS with low and high intensity.

**Figure 5** shows the topographical distribution of beta band power changes relative to the baseline (REST) averaged over all subjects for all experimental conditions. The spectral power decreases for the C, FC, and CP electrodes were summarized in **Table 2**. The scalp maps revealed power changes with a similar topography as observed in the alpha band. However, reduction of alpha band power (**Table 1**) was stronger than the reduction of beta band power (**Table 2**) in all of the experimental conditions. Execution of the MT_HIGH_ (**Figure 5A**) was again associated with the strongest ERD among all conditions, covering bilateral sensorimotor cortices with some dissemination toward centro-parietal and frontal areas. Execution of MT_LOW_ was characterized by an even less prominent ERD covering the contralateral motor cortex. Scalp maps of SS (**Figure 5B**) were characterized by a peak ERD around C3 during SS_LOW_ and SS_HIGH_ and revealed no apparent differences between the stimulation intensities. However, in comparison to the alpha band power changes, beta ERD occurred more lateralized around the contralateral hemisphere.

**Table 2:** Decrease in beta power in sensorimotor electrodes given as relative change (%) from baseline (REST).

	Contralateral			Ipsilateral		
		
Electrode	C3	CP3	FC3	C4	CP4	FC4
MT_LOW_	8.5	6.0	4.1	5.2	6.0	5.9
MT_HIGH_	13.7	11.0	6.6	11.5	9.0	9.6
SS_LOW_	7.4	7.3	4.2	4.5	5.0	2.0
SS_HIGH_	8.7	7.1	4.6	5.0	5.2	1.2


Execution of the MT_LOW_ lead to a significant reduction of beta band power in electrode C3 (*p* < 0.05) compared to REST. In contrast, execution of the MT_HIGH_ lead to a significant reduction of beta band power in FC1 (*p* < 0.001), FCz (*p* < 0.001), FC2 (*p* < 0.01) FC4 (*p* < 0.05), C3 (*p* < 0.001), C1 (*p* < 0.01), Cz (*p* < 0.05), C2 (*p* < 0.05), C4 (*p* < 0.01), CP3 (*p* < 0.001), and CP4 (*p* < 0.05). Electrode CP1 (*p* = 0.08) indicated a trend. During SS_HIGH_, significant reduction of beta band power was only observed in electrode C3 (*p* < 0.05), indicating a trend during SS_LOW_ (*p* = 0.09). Furthermore, electrode CP3 indicated a trend during SS_LOW_ (*p* = 0.051) and SS_HIGH_ (*p* = 0.055). Additionally, electrodes C1 and P3 indicated a trend (*p* = 0.08 and *p* = 0.07, respectively) during SS_HIGH_.

Similar to the alpha band power changes, we found no significant differences in beta band power between both MT conditions (**Figure 5A**) and no significant differences between both SS conditions (**Figure 5B**) in all of the 20 bilateral sensorimotor electrodes that were included in the analysis. The comparison of power changes of MT_LOW+HIGH_ vs. _SSLOW+HIGH_ revealed no significant differences during execution of the MT compared to SS. However, a trend was revealed in electrodes FC2 (*p* = 0.09), FC4 (*p* = 0.09) and CP1 (*p* = 0.1).

### Single-Trial Classification

**FIGURE 6:**
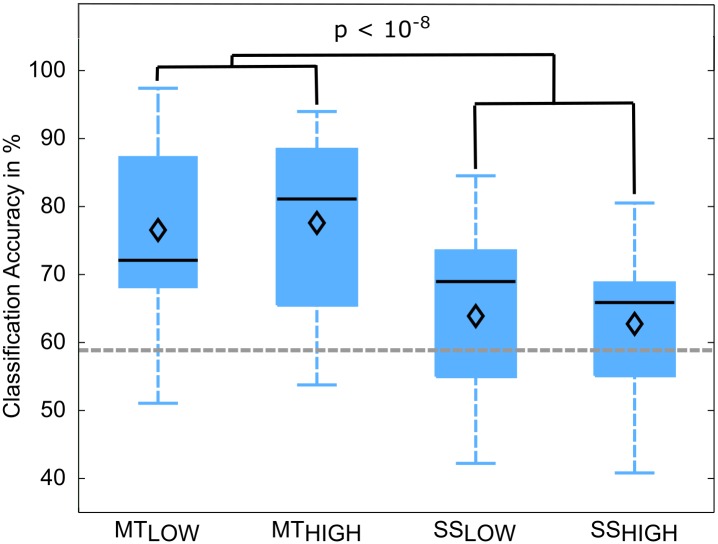
Box plots depicting the classification accuracies of the four regular classifiers (RC) using alpha band features averaged across all subjects. All classifiers performed a binary classification of an experimental condition vs. rest. Classifiers were trained and tested using a five-fold cross-validation. Each of these classifiers performed significantly above chance. MT classifiers reached significantly higher classification accuracies than sensory stimulation (SS) classifiers (*p* < 10^-8^). The vertical dotted gray line depicts the statistical chance level (58.9%). Mean classification accuracies are depicted by the diamond. Central lines within the boxes represent the median.

Classifications were performed with 86.5 ± 6.7 trials on average. The classification performances of the four RCs using alpha band features were averaged across all subjects (*n* = 13) and summarized in **Table 3**. The classification accuracies of these classifiers are depicted as boxplots in **Figure 6**. Performance of MT_LOW_ and MT_HIGH_ classifiers was significantly above statistical chance level with a classification accuracy of 76.5 ± 3.8% and 77.6 ± 3.7%, respectively. There was no statistical significant difference between classification accuracies of both MT classifiers (*p* = 0.97). Classification with SS_LOW_ and SS_HIGH_ classifiers exceeded statistical chance level with average classification accuracies of 63.9 ± 3.6% and 62.7 ± 3.4%, respectively. There was also no significant difference between classification accuracy of the SS_LOW_ and SS_HIGH_ classifiers (*p* = 0.96). However, SS classifiers performed significantly worse than MT classifiers (*p* < 10^-8^).

**Table 3:** Confusion matrices summarizing the classification performance of the four regular classifiers (RC) using alpha band features averaged over all subjects (*n* = 13).

		Prediction				
		
		MT_LOW_	REST		SS_LOW_	REST
	MT_LOW_	35.5	8.4	SS_LOW_	29.9	13.7
	REST	12.1	30.5	REST	17.7	24.9
	
**Actual**
		**MT_HIGH_**	**REST**		**SS_HIGH_**	**REST**
	
	MT_HIGH_	36.7	7.7	SS_HIGH_	28.5	15.3
	REST	12.0	30.6	REST	17.0	25.5


**FIGURE 7:**
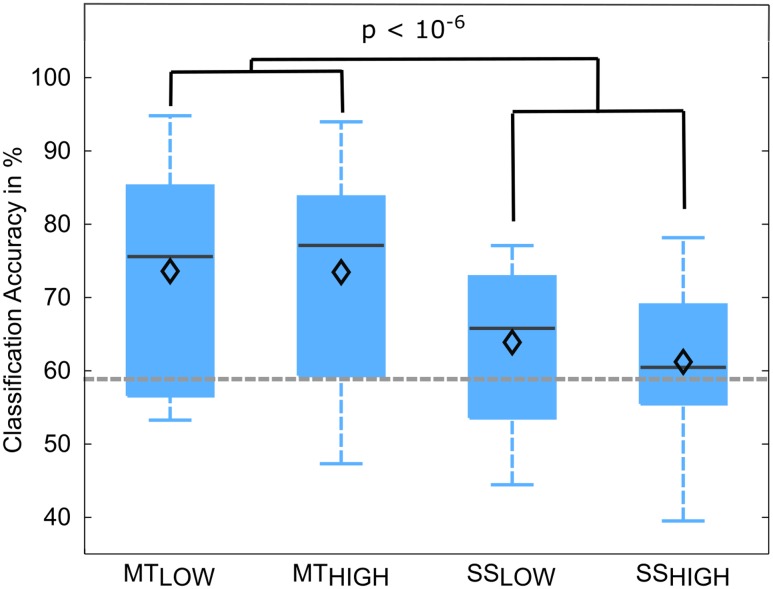
Box plots depicting the classification accuracies of the four regular classifiers (RC) using alpha and beta band features averaged across all subjects. All classifiers performed a binary classification of an experimental condition vs. rest. Classifiers were trained and tested using a five-fold cross-validation. Each of these classifiers performed significantly above chance. MT classifiers reached significantly higher classification accuracies than sensory stimulation (SS) classifiers (*p* < 10^-6^). The vertical dotted gray line depicts the statistical chance level (58.9%). Mean classification accuracies are depicted by the diamond. Central lines within the boxes represent the median.

The classification performances of the RCs using alpha and beta band features were averaged over all subjects and summarized in **Table 4**. The classification accuracies are depicted as boxplots in **Figure 7**. In detail, MT_LOW_ and MT_HIGH_ classifiers performed significantly above chance level with a classification accuracy of 73.6 ± 4.1% and 73.5 ± 4.7%. The SS_LOW_ and SS_HIGH_ classifiers exceeded chance level with classification accuracies of 63.9 ± 3.1% and 61.2 ± 3.2%, respectively. Thus, classification using alpha band and beta band features revealed very similar results to the classification using alpha band features only. There were no significant differences in classification accuracy between both MT classifiers (*p* = 0.99) and both SS classifiers (*p* = 0.68). Furthermore, SS classifiers performed again significantly worse than MT classifiers (*p* < 10^-6^).

**Table 4:** Confusion matrices summarizing the classification performance of the four regular classifiers (RC) using alpha and beta band features averaged over all subjects (*n* = 13).

		Prediction				
		
		MT_LOW_	REST		SS_LOW_	REST
	MT_LOW_	33.7	10.2	SS_LOW_	28.4	15.2
	REST	12.8	29.8	REST	16.1	26.5
	
**Actual**
		**MT_HIGH_**	**REST**		**SS_HIGH_**	**REST**
	
	MT_HIGH_	34.4	10.0	SS_HIGH_	27.1	16.8
	REST	13.3	29.3	REST	16.8	25.9


**FIGURE 8:**
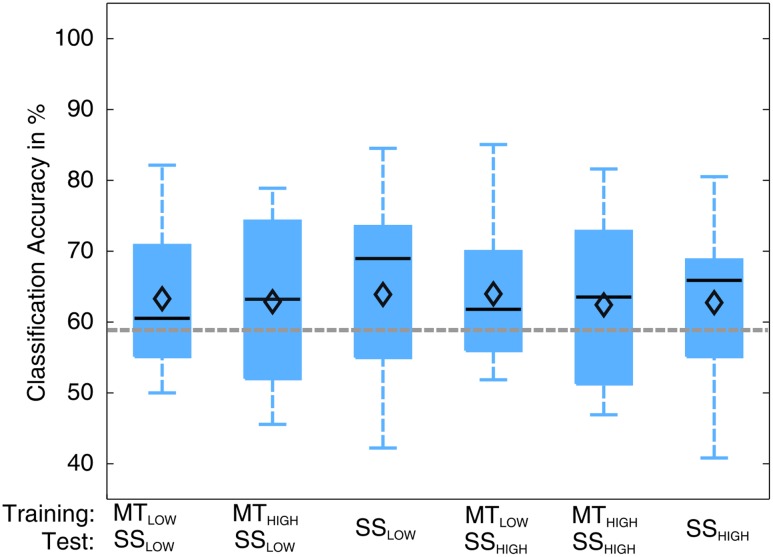
Box plots depicting the results of the cross-classification (CC) using sensory stimulation (SS) trials as input for classifiers trained on the classification of MT trials. The abscissa shows the classifications that were performed. The upper row denotes the trials that were used in the training step; the lower row denotes the trials that were used in the testing step, i.e., evaluation of the classification performance. Third and sixth boxplots depict the regular SS classifiers that serve as controls. All CCs performed significantly above chance level (*p* < 0.05). There was no significant difference in performance of any of these classifications (*p* = 0.97). CCs were able to perform classification as good as the regular classifiers (RCs) that were specifically trained to distinguish SS trials from rest trials. The vertical dotted gray line depicts the statistical chance level (58.9%). Mean classification accuracies are depicted by the diamond. Central lines within the boxes represent the median.

**FIGURE 9:**
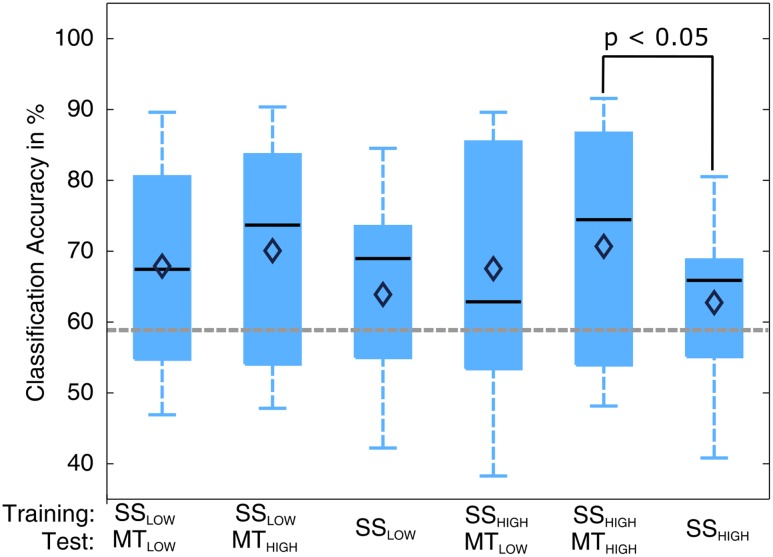
Box plots depicting the results of the cross-classification (CC) using MT trials as input for classifiers trained on the classification of sensory stimulation (SS) trials. Upper row denotes the trials that were used in the training step; lower row denotes the trials that were used in the testing step, i.e., evaluation of the classification performance. All CCs performed significantly above chance (*p* < 0.05). The CCs trained on SS and tested with MT trials reached higher classification accuracies than the regular SS classifiers (third and sixth). The CC trained on SS_HIGH_ (high intensity of SS) and tested on MT_HIGH_ (high force in the MT) even performed significantly better than the regular SS_HIGH_ classifier. The classification accuracy of the CC trained on SS_LOW_ (low intensity of sensory stimulation) and tested on MT_HIGH_ was not significantly higher than the regular SS_HIGH_ classifier but indicated a trend (*p* < 0.09). The vertical dotted gray line depicts the statistical chance level (58.9%). Mean classification accuracies are depicted by the diamond. Central lines within the boxes represent the median.

The classification performances of the CCs trained on MT and tested with SS were averaged (across all subjects) and summarized in **Table 5**. The classification accuracies are depicted as boxplots in **Figure 8**. All CCs reached classification accuracies significantly above chance (*p* < 0.05). There was no significant difference in classification accuracy of these CCs in comparison to the regular SS classifiers. The classification accuracies for the CCs MT_LOW_/SS_LOW_ and MT_HIGH_/SS_LOW_ were 63.3 ± 2.8% and 62.9 ± 3.2%, respectively. The CCs MT_LOW_/SS_HIGH_ and MT_HIGH_/SS_HIGH_ were exceeding statistical chance level with classification accuracies of 64.0 ± 2.6% and 62.4 ± 3.3%. Thus, cross-classification using SS as test set for classifiers trained on MT resulted in classification accuracies as good as the RCs that were specifically trained to distinguish SS vs. REST. The classification accuracy of the CCs trained on SS and tested with MT were averaged (across all subjects) and summarized in **Table 6**. The classification accuracies are depicted as boxplots in **Figure 9**. All CCs classifiers reached classification accuracies significantly above chance (*p* < 0.05). Interestingly, all CCs classifiers reached higher classification accuracies than the regular SS classifiers. In detail, the classification accuracies for the CCs SS_LOW_/MT_LOW_ and SS_HIGH_/MT_LOW_ were 67.9 ± 1.1% and 67.5 ± 1.3%, respectively. The cross-classifiers SS_LOW_/MT_HIGH_ and SS_HIGH_/MT_HIGH_ were exceeding statistical chance level with classification accuracies of 70.1 ± 1.2% and 70.7 ± 1.3%, respectively. Thus, cross-classification using MT as test set for classifiers trained on SS resulted in even better classification accuracies than the RCs that were specifically trained to distinguish SS vs. REST.

**Table 5:** Confusion matrices summarizing the classification performance of the cross-classifiers (CC) using alpha band features, trained on MT and tested with SS averaged over all subjects (*n* = 13).

		Prediction				
		
		MT_LOW_	REST		MT_LOW_	REST
		SS_LOW_			SS_HIGH_
	MT_LOW_	28.5	15.0	MT_LOW_	29.3	14.5
	SS_LOW_			SS_HIGH_
	REST	16.5	26.2	REST	16.5	26.2
	
**Actual**
		**MT_HIGH_**	**REST**		**MT_HIGH_**	**REST**
		**SS_LOW_**			**SS_HIGH_**
	
	MT_HIGH_	27.5	16.0	MT_HIGH_	27.3	16.5
	SS_LOW_			SS_HIGH_
	REST	15.9	26.7	REST	15.9	26.7


**Table 6:** Confusion matrices summarizing the classification performance of the cross-classifiers (CC) using alpha band features, trained on SS and tested with MT averaged over all subjects (*n* = 13).

		Prediction				
		
		SS_LOW_	REST		SS_HIGH_	REST
		MT_LOW_			MT_LOW_
	SS_LOW_	30.5	13.4	SS_HIGH_	30.8	13.0
	MT_LOW_			MT_LOW_	
	REST	14.6	28.0	REST	15.2	27.5
	
**Actual**
		**SS_LOW_**	**REST**		** SS_HIGH_**	**REST**
		**MT_HIGH_**			**MT_HIGH_**
	
	SS_LOW_	32.7	11.7	SS_HIGH_	33.8	10.6
	MT_HIGH_			MT_HIGH_	
	REST	14.6	28.0	REST	15.2	27.5


There was a significant difference in classification accuracy between the SS_HIGH_/MT_HIGH_ classifier and the regular SS_HIGH_ classifier (*p* < 0.05). The difference in classification accuracy between the SS_LOW_/MT_HIGH_ classifier and the regular SS_LOW_ classifier only indicated a trend and was not statistically significant (*p* = 0.09). Thus, the classifiers trained on SS and tested with MT resulted in even better classification accuracies than the regular SS classifiers. However, a comparison to the RCs (**Figure 5**) also revealed that they still performed significantly worse than the regular MT classifiers (*p* < 0.05).

## Discussion

### Spatial Activation Pattern

Within this study, it was investigated if the activation of the motor cortex leads to a modulation of the mu rhythm that is different from the modulation of the mu rhythm caused by the processing of sensory feedback in the sensory cortex. The MT provided an experimental setting in which the subjects perceived sensory feedback and adapted their motor output (isometric grip) to a predefined force level accordingly. A SS of the fingertips was performed to simulate the superficial touch perception in a reproducible and standardized way and the associated afferent feedback that was perceived during performance of MT without contemporaneous generation of efferent control and activation of the motor cortex.

The analysis revealed that the modulation of the mu rhythm (8-13 Hz) during execution of the MT and SS resembled each other to a very high degree. In both conditions, we observed a strong mu ERD localized around the standard sensorimotor electrodes, including the contralateral (C3) and ipsilateral sensorimotor cortex (C4) with a strong dissemination to centro-parietal cortex (CP3 and CP4). A less prominent ERD was observed bilaterally in the fronto-central electrodes (FC3 and FC4). These results are in line with previous studies that reported bilateral mu ERD during performance of various upper extremity movement-related tasks (Pfurtscheller and Neuper, 1994; Salmelin and Hari, 1994; McFarland et al., 2000). Likewise, bilateral mu ERD has been observed during pure somatosensory stimulation, e.g., electrical stimulation of the median nerve (Nikouline et al., 2000) or tactile stimulation of the index finger (Pfurtscheller, 1989; Cheyne et al., 2003). Furthermore, we discovered a stable ellipsoid ERD pattern, covering both sensorimotor cortices in anterior-posterior direction, consisting of a peak ERD at electrode C3/C4, followed by a smaller ERD in electrode CP3/CP4 and FC3/FC4 in the contralateral/ipsilateral hemisphere that was preserved in all experimental conditions.

The activation of the motor cortex during execution of the MT was indicated by two changes in mu rhythm. First, while the ERD peaks were slightly more lateralized toward the contralateral sensorimotor cortex during SS (C3 > CP3 > C4 > CP4), execution of the MT was associated with an ERD that occurred more bilaterally (C3 > C4 > CP3 > CP4). However, this change might negligible, considering that the most prominent ERD peaks were still recorded in the same three central and centro-parietal electrodes (C3, CP3, and C4) in all experimental conditions. Secondly, our study showed that the mu ERD was significantly stronger during execution of the MT compared to SS in the majority of bilateral sensorimotor electrodes. Thus, while an activation of the motor cortex did not lead to a prominent change in the spatial configuration of the mu ERD when movements were executed, it was associated with a significantly stronger mu ERD in the electrodes that already captured the mu ERD caused by afferent feedback.

These findings are largely in accordance with the established neurophysiological concept that alpha rhythms indicate a state of cortical idling, disappearing when the respective cortical area becomes activated (Pfurtscheller, 1992; Ritter et al., 2009). While SS only activates the sensory cortex, execution of the MT requires an additional activation of the motor cortex as efferent commands are generated. Thus, the stronger mu ERD during executed movements likely reflects a combination of the mu ERD caused by the processing of afferent feedback and the mu ERD caused by the generation of efferent commands. This is supported by other studies reporting that an isolated activation of the motor cortex through motor imagery (McFarland et al., 2000) or an isolated activation of the sensory cortex through somatosensory stimulation (Salenius et al., 1997) lead to a mu ERD with a similar spatial topography but smaller magnitude than the mu ERD caused by a combined activation of both cortices through executed movements. However, it has to be considered that a dynamic adjustment of grip force, as demanded in the MT, relies heavily on proprioceptive sensations derived from muscle spindles rather than superficial sensibility (Riemann and Lephart, 2002; Proske and Gandevia, 2012). Thus, parts of the stronger mu ERD during execution of the MT could be attributed to the processing of proprioceptive feedback. This is another important factor likely reducing the partial contribution of the efferent commands to the observable mu ERD when sensory feedback is present.

Furthermore, the lack of distinct differences in the spatial configuration of the ERD pattern raises the question whether the mu rhythm really encodes the activation of the sensory and motor cortices in an independent and cortex-specific manner. In fact, the stable spatial configuration seems contradictory, as this suggests that the modulation of the mu rhythm is caused by one sensorimotor network that engages to a different extent in both experimental conditions rather than two functionally distinct networks that are activated independently from each other. Furthermore, the view on the alpha rhythms’ role has been extended in the past from their initial inhibitory function toward a more complex but inherent cortical mechanism involved in the organization of the brain’s processing capacities and spatiotemporal integration of distinct but interconnected brain regions (Palva and Palva, 2007; Klimesch, 2012; Quax and Tiesinga, 2015).

Therefore, it might be more reasonable to interpret the mu rhythm in this recent framework of rhythmic alpha activity, indicating the activation of a superordinate sensorimotor network involved in the spatiotemporal organization of sensorimotor processing, exhibiting an inverse relationship to the computational effort with respect to integration of (sensory) information within the entire sensorimotor cortex. By comparing the two conditions, it is apparent that the MT is a much more complex task in terms of multimodal sensorimotor integration as it requires additional incorporation of proprioceptive feedback to dynamically adjust the generation of efferent commands compared to the processing of exclusively afferent feedback. Thus, although the magnitude of the mu ERD changes significantly as the motor cortex is activated during execution of the MT, our results suggest that the effect is attributed to sensorimotor integration in the entire sensorimotor cortex rather than specifically encoding the generation of efferent commands in the motor cortex.

### Classification of Single-Trials

The classification of single-trials was performed as additional more data-driven measure to investigate the problems that can be encountered when the generation of efferent commands is detected based on a modulation of the mu rhythm when afferent feedback is processed simultaneously. Overall, the results from the classification are in consistency with the observations from the spatial activation pattern. This can be explained with the complementary nature of the spatial activation patterns and the classification algorithm. In general, spatial activation pattern represent a form of encoding model that uses information about the experimental conditions to predict the brain activity. Information from individual trials are averaged and analyzed together to obtain an estimate of the associated brain activation. The classification algorithm is a decoding model and works the other way around, i.e., it uses brain activity from individual trials to estimate the associated experimental condition (Naselaris et al., 2011).

More precisely, the classification algorithm analyzes the brain activity in all individual trials of a subject to find a projection of the feature space that shows a maximal difference in means of brain activity recorded at individual electrodes between the experimental conditions and a minimal variance within each experimental condition (Lotte et al., 2007). Based on this, the algorithm develops a statistical model that allows the classification of the experimental conditions by evaluating a weighted sum of the characteristic features. The spatial activation patterns are simply a visual representation of the differences in brain activity between SS or MT and REST condition (**Figure 3**). Thus, a strong mu ERD corresponds to a large difference in recorded brain activity between the experimental conditions. Since the classification algorithm searches for a projection that maximizes the difference in mean between the conditions, the classification outcome will be largely dependent on the standard sensorimotor electrodes that recorded a large mu ERD (C3, CP3, C4, and CP4), as their strong activation corresponds to a large difference in mean, making them a good feature to predict the subject’s (sensorimotor) behavior and the corresponding experimental condition.

Indeed, the results from the classification confirm the assumptions drawn from the spatial activation pattern, emphasizing the idea of the mu rhythm with a rather unspecific role in sensorimotor processing. As example, all CC trained on MT and tested with SS reached classification accuracies significantly above chance (**Figure 6**). From a neurophysiological point of view, this result seems odd, considering that SS only reflects brain activity during processing of sensory feedback without the generation of efferent motor commands. If the classification of MT trials was in some way dependent on brain activity that originates during generation of efferent commands, the MT classifiers should fail to classify SS trials significantly above chance as they do not contain efferent commands. However, the analysis of the activation pattern already revealed the lack of major differences in the spatial configuration between execution of the MT and SS. Instead, both were characterized by peak ERDs in the same bilateral sensorimotor electrodes. Thus, with respect to the mathematical working principle of the classification algorithm described above, it is reasonable to deduce that the classifiers, whether they are trained on MT or SS, always strongly rely on these four sensorimotor electrodes when deciding to which experimental condition a trial belongs. As consequence, cross-classification worked well even though SS does not contain any efferent commands.

Conversely, classifiers trained on SS were also able to classify MT trials significantly above chance. This is indeed explainable from a neurophysiological point of view, considering that SS and MT both contain brain activity associated with the processing of afferent sensory feedback. However, all classifiers reached higher classification accuracies than the regular SS classifiers, even significantly higher for the SS_HIGH_/MT_HIGH_ classifier and indicting a trend for the SS_LOW_/MT_HIGH_ classifier. This higher classification accuracy is attributed to the stronger brain activation represented by a significantly lower averaged amplitude of nearly all alpha band frequency bins during execution of the MT. Since the SS classifier weights the same electrodes as a MT classifier, the stronger activation during execution of the MT simply makes it easier for the classifier to decide to which experimental condition a trial belongs. Indeed, the RC already revealed a significantly better classification performance of MT classifiers, indicating that the stronger brain activity during motor execution permits a better distinction vs. REST in comparison to the SS trials. Thus, these results strongly support the hypothesis that the classifiers always evaluate the same electrodes with similar weights when assigning a trial to a condition. This hypothesis would also explain why the addition of beta band features did not further increase the classification accuracy during regular classification. The spatial pattern already indicated that the changes in beta power were relatively small compared to the alpha power changes. Thus, when the classifier finds a projection that maximizes the difference in class means, the alpha band features from these four electrodes will largely drive this process whereas the beta band features might have little impact. However, it should be clarified that this does not imply that the beta band does not contain any useful information for the detection of motor commands in general. Instead, the results rather demonstrate that small changes in beta oscillations might only play a minor role for classification in the presence of stronger alpha band changes even if those are not truly specific for the experimental condition itself, leading to a classifier that is based on the most prominent, but maybe “wrong” pattern. Here, this “wrong” pattern corresponds to the mu ERD that shows a similar topographical pattern with similar amplitudes during different sensorimotor conditions even though the modulation does not seem to be specific for the generation of efferent commands.

Furthermore, studies have shown that motor imagery is represented by similar neural substrates as motor execution but the associated brain activity is around ∼25–30% smaller in magnitude (Porro et al., 1996; Miller et al., 2010). Based on our results, it can be expected that a motor imagery based detection of motor intention and control are even more prone to a false positive detection caused by sensory feedback as both processes are not only characterized by similar spatial patterns but also likely match in magnitude. However, this hypothesis needs to be confirmed in future studies using motor imagery. The results of this study are limited to some extent, as three of the study participants (S1, S7, and S8) did not show any apparent patterns around the sensorimotor cortex in relation to the experimental conditions likely reducing the significance of the results. As a consequence, classification accuracies only exceeded statistical chance level in at best one of the four regular classifiers in these subjects. However, this finding is in line with Nombela and Nombela (2013), demonstrating that the mu rhythm only appears in 76% of the able-bodied population. Furthermore, EEG source localization algorithms might give additional information about the cortical origin of the mu ERD and provide further insights in the underlying motor and/or sensory-related processes. However, these algorithms are computationally demanding and not real-time compatible. Thus, their benefit might be quite limited for rehabilitative technologies that use BCI-based detection of motor commands in the attempt to re-establish a functional sensorimotor loop to promote motor learning.

## Conclusion

Overall, our results demonstrate some of the jeopardies that can be encountered when the mu rhythm is used to detect efferent motor commands. We found that different aspects of sensorimotor processing are characterized by identical spatial pattern of the mu rhythm, even if decisive factors like the activation of the motor cortex during generation of efferent motor commands are absent. Thus, mu rhythm based detection of efferent motor commands is highly susceptible to a false positives caused by the processing of afferent sensory feedback.

## Author Contributions

MH designed the study, performed parts of the experimental work, data analysis and wrote main parts of the article. MS gave major technical support and conceptual advice for the study design. CS performed the statistical analysis and developed programming scripts for data recording and analysis. PK was involved in the experimental work and wrote parts of the manuscript. RR supervised the complete design of the project, provided major help in analyzing the data, revised the manuscript for publication and applied for ethical approval.

## Conflict of Interest Statement

The authors declare that the research was conducted in the absence of any commercial or financial relationships that could be construed as a potential conflict of interest.

## References

[B1] Ang K. K. Guan C. Chua K. S. Ang B. T. Kuah C. Wang C. (2010). Clinical study of neurorehabilitation in stroke using EEG-based motor imagery brain-computer interface with robotic feedback. *Conf. Proc. IEEE Eng. Med. Biol. Soc.* 2010 5549–5552. 10.1109/IEMBS.2010.5626782 21096475

[B2] Arroyo S. Lesser R. P. Gordon B. Uematsu S. Jackson D. Webber R. (1993). Functional significance of the mu rhythm of human cortex: an electrophysiologic study with subdural electrodes. *Electroencephalogr. Clin. Neurophysiol.* 87 76–87. 10.1016/0013-4694(93)90114-B 7691544

[B3] Babiloni C. Carducci F. Cincotti F. Rossini P. M. Neuper C. Pfurtscheller G. (1999). Human movement-related potentials vs desynchronization of EEG alpha rhythm: a high-resolution EEG study. *Neuroimage* 10 658–665. 10.1006/nimg.1999.0504 10600411

[B4] Cauraugh J. H. Summers J. J. (2005). Neural plasticity and bilateral movements: a rehabilitation approach for chronic stroke. *Prog. Neurobiol.* 75 309–320. 10.1016/j.pneurobio.2005.04.001 15885874

[B5] Cheyne D. Gaetz W. Garnero L. Lachaux J. P. Ducorps A. Schwartz D. (2003). Neuromagnetic imaging of cortical oscillations accompanying tactile stimulation. *Brain Res. Cogn. Brain Res.* 17 599–611. 10.1016/S0926-6410(03)00173-3 14561448

[B6] Cole J. (1995). *Pride and a Daily Marathon.* Cambridge, MA: MIT Press.

[B7] Collinger J. L. Boninger M. L. Bruns T. M. Curley K. Wang W. Weber D. J. (2013). Functional priorities, assistive technology, and brain-computer interfaces after spinal cord injury. *J. Rehabil. Res. Dev.* 50 145–160. 10.1682/JRRD.2011.11.0213 23760996 PMC3684986

[B8] Combrisson E. Jerbi K. (2015). Exceeding chance level by chance: the caveat of theoretical chance levels in brain signal classification and statistical assessment of decoding accuracy. *J. Neurosci. Methods* 250 126–136. 10.1016/j.jneumeth.2015.01.010 25596422

[B9] Daly J. J. Wolpaw J. R. (2008). Brain-computer interfaces in neurological rehabilitation. *Lancet Neurol.* 7 1032–1043. 10.1016/S1474-4422(08)70223-0 18835541

[B10] Fisher R. A. (1936). The use of multiple measurements in taxonomic problems. *Ann. Eugen.* 7 179–188. 10.1111/j.1469-1809.1936.tb02137.x

[B11] Galán F. Baker M. R. Alter K. Baker S. N. (2015). Degraded EEG decoding of wrist movements in absence of kinaesthetic feedback. *Hum. Brain Mapp.* 36 643–654. 10.1002/hbm.22653 25307551 PMC4312958

[B12] Galan F. Nuttin M. Lew E. Ferrez P. W. Vanacker G. Philips J. (2008). A brain-actuated wheelchair: asynchronous and non-invasive Brain-computer interfaces for continuous control of robots. *Clin. Neurophysiol.* 119 2159–2169. 10.1016/j.clinph.2008.06.001 18621580

[B13] Ghez C. Gordon J. Ghilardi M. F. (1995). Impairments of reaching movements in patients without proprioception. II. Effects of visual information on accuracy. *J. Neurophysiol.* 73 361–372. 7714578 10.1152/jn.1995.73.1.361

[B14] Gomez-Rodriguez M. Peters J. Hill J. Scholkopf B. Gharabaghi A. Grosse-Wentrup M. (2011). Closing the sensorimotor loop: haptic feedback facilitates decoding of motor imagery. *J. Neural Eng.* 8:036005. 10.1088/1741-2560/8/3/036005 21474878

[B15] Grefkes C. Ward N. S. (2014). Cortical reorganization after stroke: how much and how functional? *Neuroscientist* 20 56–70. 10.1177/1073858413491147 23774218

[B16] Hallett M. (2001). Plasticity of the human motor cortex and recovery from stroke. *Brain Res. Brain Res. Rev.* 36 169–174. 10.1016/S0165-0173(01)00092-3 11690613

[B17] Jensen T. R. Radwin R. G. Webster J. G. (1991). A conductive polymer sensor for measuring external finger forces. *J. Biomech.* 24 851–858. 10.1016/0021-9290(91)90310-J 1752869

[B18] Kilavik B. E. Zaepffel M. Brovelli A. MacKay W. A. Riehle A. (2013). The ups and downs of beta oscillations in sensorimotor cortex. *Exp. Neurol.* 245 15–26. 10.1016/j.expneurol.2012.09.014 23022918

[B19] Klimesch W. (2012). Alpha-band oscillations, attention, and controlled access to stored information. *Trends Cogn. Sci.* 16 606–617. 10.1016/j.tics.2012.10.007 23141428 PMC3507158

[B20] Krakauer J. W. (2006). Motor learning: its relevance to stroke recovery and neurorehabilitation. *Curr. Opin. Neurol.* 19 84–90. 10.1097/01.wco.0000200544.29915.cc 16415682

[B21] Kubler A. Neumann N. Kaiser J. Kotchoubey B. Hinterberger T. Birbaumer N. P. (2001). Brain-computer communication: self-regulation of slow cortical potentials for verbal communication. *Arch. Phys. Med. Rehabil.* 82 1533–1539. 10.1053/apmr.2001.26621 11689972

[B22] Kuhlman W. N. (1978). Functional topography of the human mu rhythm. *Electroencephalogr. Clin. Neurophysiol.* 44 83–93. 10.1016/0013-4694(78)90107-4 74329

[B23] Lotte F. Congedo M. Lécuyer A. Lamarche F. Arnaldi B. (2007). A review of classification algorithms for EEG-based brain–computer interfaces. *J. Neural Eng.* 4 R1–R13. 10.1088/1741-2560/4/2/R01 17409472

[B24] McFarland D. J. McCane L. M. David S. V. Wolpaw J. R. (1997). Spatial filter selection for EEG-based communication. *Electroencephalogr. Clin. Neurophysiol.* 103 386–394. 10.1016/S0013-4694(97)00022-2 9305287

[B25] McFarland D. J. Miner L. A. Vaughan T. M. Wolpaw J. R. (2000). Mu and beta rhythm topographies during motor imagery and actual movements. *Brain Topogr.* 12 177–186. 10.1023/A:1023437823106 10791681

[B26] Miller K. J. Schalk G. Fetz E. E. den Nijs M. Ojemann J. G. Rao R. P. (2010). Cortical activity during motor execution, motor imagery, and imagery-based online feedback. *Proc. Natl. Acad. Sci. U.S.A.* 107 4430–4435. 10.1073/pnas.0913697107 20160084 PMC2840149

[B27] Naselaris T. Kay K. N. Nishimoto S. Gallant J. L. (2011). Encoding and decoding in fMRI. *Neuroimage* 56 400–410. 10.1016/j.neuroimage.2010.07.073 20691790 PMC3037423

[B28] Nicolas-Alonso L. F. Gomez-Gil J. (2012). Brain computer interfaces, a review. *Sensors* 12 1211–1279. 10.3390/s120201211 22438708 PMC3304110

[B29] Nikouline V. V. Linkenkaer-Hansen K. Wikstrom H. Kesaniemi M. Antonova E. V. Ilmoniemi R. J. (2000). Dynamics of mu-rhythm suppression caused by median nerve stimulation: a magnetoencephalographic study in human subjects. *Neurosci. Lett.* 294 163–166. 10.1016/S0304-3940(00)01562-7 11072140

[B30] Nombela C. Nombela M. (2013). IS *MU* A NORMAL RHYTHM. *Orthop. Muscul. Syst.* 2 122 10.4172/2161-0533.1000122

[B31] Nudo R. J. Wise B. M. SiFuentes F. Milliken G. W. (1996). Neural substrates for the effects of rehabilitative training on motor recovery after ischemic infarct. *Science* 272 1791–1794. 10.1126/science.272.5269.1791 8650578

[B32] Onose G. Grozea C. Anghelescu A. Daia C. Sinescu C. J. Ciurea A. V. (2012). On the feasibility of using motor imagery EEG-based brain-computer interface in chronic tetraplegics for assistive robotic arm control: a clinical test and long-term post-trial follow-up. *Spinal Cord* 50 599–608. 10.1038/sc.2012.14 22410845

[B33] Palva S. Palva J. M. (2007). New vistas for α-frequency band oscillations. *Trends Neurosci.* 30 150–158. 10.1016/j.tins.2007.02.001 17307258

[B34] Parra L. C. Spence C. D. Gerson A. D. Sajda P. (2005). Recipes for the linear analysis of EEG. *Neuroimage* 28 326–341. 10.1016/j.neuroimage.2005.05.032 16084117

[B35] Peterka R. J. (2002). Sensorimotor integration in human postural control. *J. Neurophysiol.* 88 1097–1118. 12205132 10.1152/jn.2002.88.3.1097

[B36] Pfurtscheller G. (1989). Functional topography during sensorimotor activation studied with event-related desynchronization mapping. *J. Clin. Neurophysiol.* 6 75–84. 10.1097/00004691-198901000-00003 2915031

[B37] Pfurtscheller G. (1992). Event-related synchronization (ERS): an electrophysiological correlate of cortical areas at rest. *Electroencephalogr. Clin. Neurophysiol.* 83 62–69. 10.1016/0013-4694(92)90133-3 1376667

[B38] Pfurtscheller G. Brunner C. Schlogl A. Lopes da Silva F. H. (2006). Mu rhythm (de)synchronization and EEG single-trial classification of different motor imagery tasks. *Neuroimage* 31 153–159. 10.1016/j.neuroimage.2005.12.003 16443377

[B39] Pfurtscheller G. Lopes da Silva F. H. (1999). Event-related EEG/MEG synchronization and desynchronization: basic principles. *Clin. Neurophysiol.* 110 1842–1857. 10.1016/S1388-2457(99)00141-8 10576479

[B40] Pfurtscheller G. Muller G. R. Pfurtscheller J. Gerner H. J. Rupp R. (2003). ‘Thought’–control of functional electrical stimulation to restore hand grasp in a patient with tetraplegia. *Neurosci. Lett.* 351 33–36. 10.1016/S0304-3940(03)00947-9 14550907

[B41] Pfurtscheller G. Neuper C. (1994). Event-related synchronization of mu rhythm in the EEG over the cortical hand area in man. *Neurosci. Lett.* 174 93–96. 10.1016/0304-3940(94)90127-9 7970165

[B42] Pfurtscheller G. Stancak A. Jr. Neuper C. (1996). Event-related synchronization (ERS) in the alpha band–an electrophysiological correlate of cortical idling: a review. *Int. J. Psychophysiol.* 24 39–46. 10.1016/S0167-8760(96)00066-9 8978434

[B43] Porro C. A. Francescato M. P. Cettolo V. Diamond M. E. Baraldi P. Zuiani C. (1996). Primary motor and sensory cortex activation during motor performance and motor imagery: a functional magnetic resonance imaging study. *J. Neurosci.* 16 7688–7698. 8922425 10.1523/JNEUROSCI.16-23-07688.1996 PMC6579073

[B44] Prasad G. Herman P. Coyle D. McDonough S. Crosbie J. (2010). Applying a brain-computer interface to support motor imagery practice in people with stroke for upper limb recovery: a feasibility study. *J. Neuroeng. Rehabil.* 7:60. 10.1186/1743-0003-7-60 21156054 PMC3017056

[B45] Proske U. Gandevia S. C. (2012). The proprioceptive senses: their roles in signaling body shape, body position and movement, and muscle force. *Physiol. Rev.* 92 1651–1697. 10.1152/physrev.00048.2011 23073629

[B46] Quax S. C. Tiesinga P. (2015). Alpha phase modulates the effectiveness and directionality of cortical communication. *BMC Neurosci.* 16(Suppl. 1):P260 10.1186/1471-2202-16-S1-P260

[B47] Riemann B. L. Lephart S. M. (2002). The sensorimotor system, part II: the role of proprioception in motor control and functional joint stability. *J. Athl. Train.* 37 80–84. 16558671 PMC164312

[B48] Ritter P. Moosmann M. Villringer A. (2009). Rolandic alpha and beta EEG rhythms’ strengths are inversely related to fMRI-BOLD signal in primary somatosensory and motor cortex. *Hum. Brain Mapp.* 30 1168–1187. 10.1002/hbm.20585 18465747 PMC6870597

[B49] Rohm M. Schneiders M. Muller C. Kreilinger A. Kaiser V. Muller-Putz G. R. (2013). Hybrid brain-computer interfaces and hybrid neuroprostheses for restoration of upper limb functions in individuals with high-level spinal cord injury. *Artif. Intell. Med.* 59 133–142. 10.1016/j.artmed.2013.07.004 24064256

[B50] Salenius S. Schnitzler A. Salmelin R. Jousmaki V. Hari R. (1997). Modulation of human cortical rolandic rhythms during natural sensorimotor tasks. *Neuroimage* 5 221–228. 10.1006/nimg.1997.0261 9345551

[B51] Salmelin R. Hari R. (1994). Spatiotemporal characteristics of sensorimotor neuromagnetic rhythms related to thumb movement. *Neuroscience* 60 537–550. 10.1016/0306-4522(94)90263-1 8072694

[B52] Schnitzler A. Salmelin R. Salenius S. Jousmaki V. Hari R. (1995). Tactile information from the human hand reaches the ipsilateral primary somatosensory cortex. *Neurosci. Lett.* 200 25–28. 10.1016/0304-3940(95)12065-C 8584258

[B53] Scott S. H. (2004). Optimal feedback control and the neural basis of volitional motor control. *Nat. Rev. Neurosci.* 5 532–546. 10.1038/nrn1427 15208695

[B54] Soekadar S. R. Birbaumer N. Slutzky M. W. Cohen L. G. (2015). Brain-machine interfaces in neurorehabilitation of stroke. *Neurobiol. Dis.* 83 172–179. 10.1016/j.nbd.2014.11.025 25489973

[B55] Steingrüber H.-J. Lienert G. A. (1971). *Hand-Dominanz-Test: HDT.* Göttingen: Hogrefe Verlag für Psychologie.

[B56] Waldert S. (2016). Invasive vs. Non-invasive neuronal signals for brain-machine interfaces: will one prevail? *Front. Neurosci.* 10:295. 10.3389/fnins.2016.00295 27445666 PMC4921501

[B57] Wolpaw J. R. McFarland D. J. (2004). Control of a two-dimensional movement signal by a noninvasive brain-computer interface in humans. *Proc. Natl. Acad. Sci. U.S.A.* 101 17849–17854. 10.1073/pnas.0403504101 15585584 PMC535103

[B58] Wolpaw J. R. McFarland D. J. Neat G. W. Forneris C. A. (1991). An EEG-based brain-computer interface for cursor control. *Electroencephalogr. Clin. Neurophysiol.* 78 252–259. 10.1016/0013-4694(91)90040-B 1707798

[B59] Yuan H. He B. (2014). Brain-computer interfaces using sensorimotor rhythms: current state and future perspectives. *IEEE Trans. Biomed. Eng.* 61 1425–1435. 10.1109/TBME.2014.2312397 24759276 PMC4082720

[B60] Yuan H. Liu T. Szarkowski R. Rios C. Ashe J. He B. (2010). Negative covariation between task-related responses in alpha/beta-band activity and BOLD in human sensorimotor cortex: an EEG and fMRI study of motor imagery and movements. *Neuroimage* 49 2596–2606. 10.1016/j.neuroimage.2009.10.028 19850134 PMC2818527

[B61] Zaepffel M. Trachel R. Kilavik B. E. Brochier T. (2013). Modulations of EEG beta power during planning and execution of grasping movements. *PLOS ONE* 8:e60060. 10.1371/journal.pone.0060060 23555884 PMC3605373

